# Association between Serum Osteoprotegerin Levels and Severity of Coronary Artery Disease in Patients with Acute Myocardial Infarction

**DOI:** 10.3390/jcm10194326

**Published:** 2021-09-23

**Authors:** Yves Cottin, Rany Issa, Mourad Benalia, Basile Mouhat, Alexandre Meloux, Laura Tribouillard, Florence Bichat, Luc Rochette, Catherine Vergely, Marianne Zeller

**Affiliations:** 1 Cardiology Department, CHU Dijon Bourgogne, 21000 Dijon, France; yves.cottin@chu-dijon.fr (Y.C.); rany.issa@chu-dijon.fr (R.I.); mourad.benalia@chu-dijon.fr (M.B.); basile.mouhat@chu-dijon.fr (B.M.); laura.tribouillard@hotmail.fr (L.T.); florence.bichat@chu-dijon.fr (F.B.); 2 Equipe d’Accueil (EA 7460), Physiopathologie et Epidémiologie Cérébro-Cardiovasculaires (PEC2), Faculté des Sciences de Santé Université de Bourgogne—Franche Comté, 7 Bd Jeanne d’Arc, 21000 Dijon, France; alexandre.meloux@u-bourgogne.fr (A.M.); luc.rochette@u-bourgogne.fr (L.R.); catherine.vergely@u-bourgogne.fr (C.V.)

**Keywords:** myocardial infarction, coronary artery disease, SYNTAX score, osteoprotegerin, vascular calcification

## Abstract

Background. Osteoprotegerin (OPG), a glycoprotein of the tumour necrosis factor (TNF) superfamily, is one of the main biomarkers for vascular calcification. Aim. We aimed to evaluate the association between serum OPG levels and extent of coronary lesions in patients with acute myocardial infarction (MI). Methods. Consecutive patients hospitalized for an acute MI who underwent coronary angiography were included. SYNTAX score was calculated to assess the severity of coronary artery disease. The population was analysed in low (5 (3–6)), medium (11 (9–13)) and high (20 (18–23)) tertiles of SYNTAX score. Results. Among the 378 patients included, there was a gradual increase in age, rate of diabetes, anterior wall location, and a reduction in left ventricular ejection fraction across the SYNTAX tertiles. OPG levels significantly increased across the tertiles (962 (782–1497), 1240 (870–1707), and 1464 (1011–2129) pg/mL, respectively (*p* < 0.001)). In multivariate analysis, OPG [OR(CI95%): 2.10 (1.29–3.49) 0.003], were associated with the high SYNTAX group, beyond hypercholesterolemia, CV history and reduced glomerular filtration rate. Conclusion. We found an association between OPG levels and coronary lesions complexity patients with acute MI.

## 1. Introduction

Osteoprotegerin (OPG), a glycoprotein of the tumour necrosis factor (TNF) receptor superfamily, acts as a dummy receptor for the nuclear factor κ-B ligand receptor (RANKL) and the TNF-linked apoptosis-inducing ligand (TRAIL) [1,2]. It is expressed in vivo in bone cells (osteoblasts), vascular smooth muscle cells and endothelial cells [3,4]. It is detected by immunohistochemistry in aortic and coronary atherosclerotic plaques. OPG is known to inhibit osteoclastogenesis and animal studies have shown a protective effect [5]. Vascular calcification (VC) is an active, complex process, where the key step towards calcification is the transformation of vascular smooth muscle cells to osteoblast phenotype, subsequently leading to hydroxyapatite accumulation, and the formation of cartilage and bone in the arterial wall. Although the underlying mechanisms linking VC to atherosclerotic cardiovascular diseases (ASCVD) are largely unknown, growing evidence suggests that OPG plays a key role in the development of VC [6]. High levels of OPG have been associated with coronary artery calcification and the development of coronary artery disease (CAD) [7,8,9,10,11]. Moreover, serum OPG could be considered a useful biomarker in patients with acute or chronic cardiometabolic disease [12].

However, only a few studies have evaluated the relationship between the extent of CAD by coronary angiography and circulating levels of OPG. Jono et al. showed an association between serum OPG and the number of coronary lesions in a series of 200 patients with stable CAD [10]. However, the clinical significance of OPG levels in patients with acute MI remains unexplored.

The objective of our study was to address the relationship between serum OPG levels and the extension of CAD in patients with acute MI, as measured by the severity of the coronary lesions using the SYNTAX score.

## 2. Materials and Methods

### 2.1. Population

From the RICO survey (RICO) [13], all consecutive patients admitted in the coronary care unit of Dijon University Hospital for an acute MI from May 2017 to February 2018 and who underwent coronary angiography were prospectively included. Patients < 18 y, with prior AF (paroxysmal or permanent), type 2 MI or time delay from symptom onset to admission > 12H were excluded from the study. MI was defined according to current criteria of European Society of Cardiology (ESC) [14,15]. All of the participants provided consent prior to inclusion, and the Ethics Committee of the University Hospital of Dijon approved the protocol (BIOCARDIS-2016–9205AAO034S02117).

### 2.2. Data Collection

CV risk factors were collected, i.e., age, gender, hypertension, diabetes, obesity (body mass index (BMI)), prior high cholesterol or total cholesterol > 2.5 g/L, familial history of CAD (premature CAD in a first-degree relative < 55 years of age for men or <65 years of age for women) and current smoking.

ASCVD history (CAD, stroke, carotid atheroma or peripheral arterial disease (PAD)), chronic renal failure or congestive heart failure were also collected. Systolic and diastolic blood pressure (SBP and DBP), heart rate (HR) were measured on admission. Biological data were obtained from blood sampling on admission (blood glucose, CRP, NT-pro-BNP, creatinine and estimated Glomerular Filtration Rate (CKD-EPI). Troponin Ic peak was obtained from 3 blood samples every 8 h after admission. Left ventricular ejection fraction (LVEF) was measured within 24 h using the Simpson biplane method [16]. GRACE risk score was also calculated [17]. In-hospital events including CV mortality, heart failure (as defined as Killip class >2) and recurrent MI were collected.

### 2.3. Serum OPG Level Assessment

Blood samples were taken upon admission to assess circulating OPG levels from radial or femoral artery in a non-heparinized test tube. Blood samples were stored at +4 °C for up to 24 h. Stability of the biomarker in preclinical conditions has been tested in healthy volunteers (*n* = 10) and in MI patients (*n* = 6). The samples were centrifuged and stored at −80 °C before analysis. For each assay, a pooled sample from 3 healthy volunteers was used as a control. Serum OPG was measured in duplicate using a commercially available multiplex assay (Human Magnetic Luminex Assay, R&D Systems—Bio-Techne, Lille, France), according to manufacturer instructions. The detection limit of this assay was (25 pg/mL) and both the intra-and interassay coefficients of variation were < 10%. 

### 2.4. Coronary Angiography

Coronary angiography images were reviewed blinded (without knowledge of the patient’s group) by two trained interventional cardiologists. A discrepancy was noted for 7 cases, for which a joint review allowed the final adjudication. SYNTAX anatomical score, before PCI was calculated to address CAD extent and coronary lesions’ complexity (length, bifurcation, diffuse disease, calcification, thrombus, total occlusion) [17]. 

### 2.5. Statistical Analysis

Dichotomous variables were expressed as *n* (%) and continuous variables in median (interquartile range (IQR)). The normality of the variables was tested by the Kolmogorov–Smirnoff test. For categorical data, a chi-square or Fischer’s exact test and a Student’s test or a Mann–Whitney test for continuous data were used, as appropriate. The significance threshold was set at 5%. A multivariate logistic regression analysis was performed to estimate high SYNTAX score (last tertile), including variables significantly associated in univariate analysis, with an inclusion and exclusion threshold set at 5% (Age > 65 year, hypertension, diabetes, hypercholesterolemia, prior CAD, GFR < 60 mL/min/1.73 m^2^). The optimal OPG threshold (>1080 pg/mL) for predicting a high SYNTAX score was obtained by the ROC (receiver–operation characteristic) curve analysis (AUC 0.60, *p* < 0.001) and Youden’s index (sensitivity: 58%; specificity: 70%).

## 3. Results

**Figure 1:**
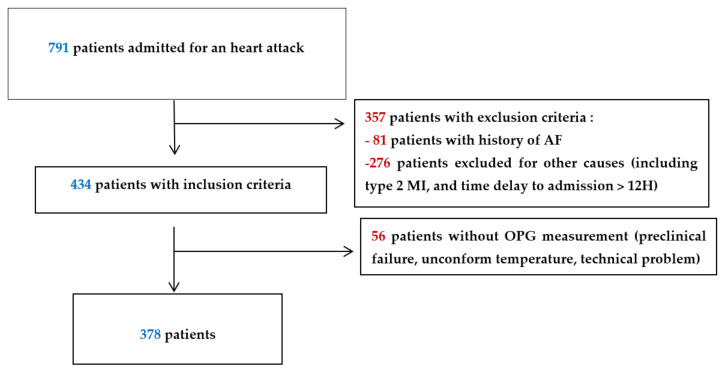
Flow chart.

Of the 791 consecutive patients admitted for a heart attack during the inclusion period, 378 were analysed (Figure 1). 

Baseline characteristics are presented on SYNTAX score tertiles, ranging from low (5(3–6)), to medium (11(9–13)) and high (20(18–23)) SYNTAX score (Table 1). Across the tertiles, there was a gradual increase in age, rate of diabetes, anterior wall location and GRACE risk score, while LVEF was gradually reduced. The rate of STEMI/NSTEMI was similar across the tertiles (*p* = 0.520). Patients from the highest SYNTAX tertile had more prior ASCVD and CABG than in lower tertiles. In addition, hospital CV events were more frequent in the highest SYNTAX group (Table 1).

**Figure 2:**
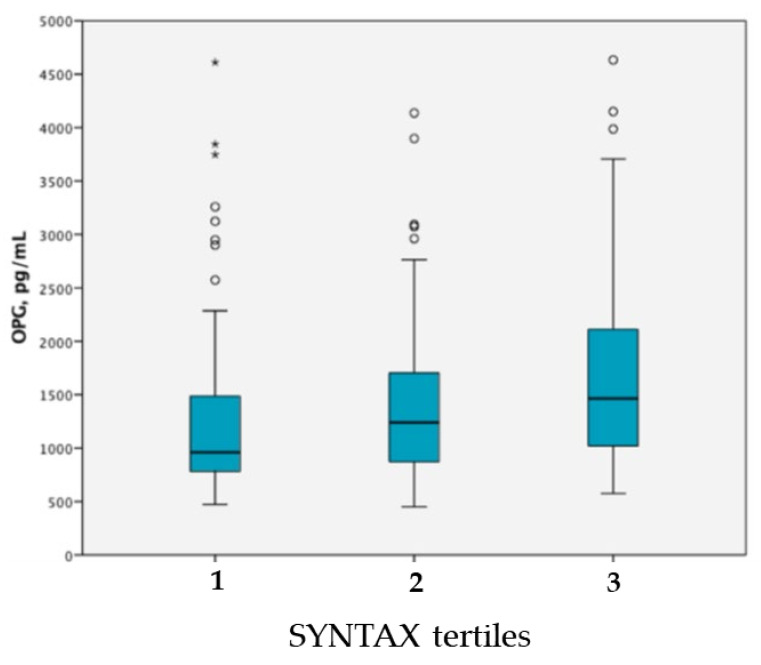
Median OPG levels according to SYNTAX score (*p* < 0.001). Circle: outlier; *: extreme oulier.

Patients with more severe CAD extent (Tertile 3) had higher levels of OPG (*p* < 0.001) (Figure 2) and rates of patients with high OPG (>1080 pg/mL) gradually increased across the tertiles (Table 2).

Patients with a high SYNTAX score had also elevated creatinine (*p* < 0.001), and NT-pro-BNP levels (*p* < 0.001). However, the Troponin Ic peak was similar for the three groups. 

In multivariate analysis, a high OPG level was the strongest factor associated with high CAD extent (OR: 2.09(1.31–3.32)), beyond hypercholesterolemia, prior CAD and reduced GFR (Table 3). 

## 4. Discussion

Our work on a large prospective study in acute MI highlights that circulating levels of OPG are associated with the severity of CAD, as assessed by the SYNTAX score. 

Vascular calcifications (VC) are typical features of advanced ASCVD, and OPG, as a bone key for atherosclerosis, is associated with disease progression. Moreover, patients with VC are at higher risk for CV events [18]. A growing body of evidence suggests that the triad OPG and its ligands (RANKL and TRAIL) exert pro-atherogenic and pro-diabetogenic effects by amplifying the adverse effects of inflammation [19]. Moreover, an increase in serum OPG levels had been shown to be predictor of CV mortality in patients with chronic coronary syndrome [20,21]. A 10-year follow-up survey showed that serum OPG levels were an independent risk factor for the progression of atherosclerosis, CV events and mortality [22]. Finally, OPG has been shown to predict early carotid atherosclerosis in patients with CAD [23,24].

### 4.1. OPG Is Associated with the Severity of Coronary Artery Disease 

Serum OPG levels increased with the number of lesions on coronary angiography in 200 patients with CAD [11]. More recently, serum OPG levels correlated with CAD severity by angiographic Gensini score, and CV mortality was higher at midterm follow-up in patients with high OPG levels [25]. In our study, we further showed a strong association between the degree of complexity of the coronary lesions by SYNTAX score and the OPG level in patients with acute MI. In contrast, in a prior study including 40 patients, no significant association had been found between serum OPG levels and elevated SYNTAX scores [13]. However, the small sample size and the high number of exclusion criteria weaken the conclusions drawn from this work.

Our work showed a strong and independent relationship between OPG levels and the severity of CAD in acute MI. Therefore, this biomarker, which is easily measured with blood sampling on admission, could help in predicting the severity of CAD.

### 4.2. OPG as a New Biomarker in Coronary Artery Disease 

We showed that patients with elevated serum OPG (≥1080 pg/mL) were more likely to have a high SYNTAX score. An upper limit of OPG at 1412 pg/mL with an AUC of 0.60 (*p* = 0.028) was found to be related with the calcium score in patients with chronic kidney disease [25]. Moreover, a significant association between the OPG level and a calcium score >100, found an OPG cut-off value of 953 pg/mL with a sensitivity of 62% and a specificity of 82% [26]. These thresholds, close to those of our current work, could be proposed as a reference limit beyond which we could consider ischemia testing in patients at high CV risk. In agreement with the literature, we found that CV risk factors, such as hypercholesterolemia, CV history and elevated creatinine level, were strong predictors of extensive CAD [27,28,29]. Given the potential therapeutic strategies targeting VC, further studies are thus needed to better understand the place of OPG as a biomarker in ASCVD [30].

### 4.3. Study Limitations

This study has several limitations. This was a monocentric study and our findings may not be extrapolated to other MI populations. Moreover, coronary calcium score, a reliable surrogate marker of atherosclerosis, was not available in our study. However, characteristics including the median age (63 years) and the rate of main CV risk factors are similar to those found in MI registries, strongly suggesting their representativeness [31]. Finally, pre-analytical variables may influence OPG measurements. However, to reduce variability within the process, all the pre-analytical and analytical conditions were similar for all study participants, as recommended [32]. 

## 5. Conclusions

In a prospective study on patients with acute MI, we found an association between serum OPG levels and CAD burden, as assessed by the SYNTAX score. Further multicentre studies are needed to confirm the relevance of the determination of OPG levels in patients with acute MI.

## Figures and Tables

**Table 1:** Baseline characteristics, according to SYNTAX score tertiles. *n*(%) or median (IQR).

		SYNTAX Score		
	Tertile 1	Tertile 2	Tertile 3	*p*
	N = 125	N = 128	N = 125	
**SYNTAX score**	5 (3–6)	11 (9–13)	20 (18–23)	<0.001
**Min_max**	2–7	8–16	17–36	
**Risk factors**				
**Age (year)**	58 (53–71)	64 (56–74)	69 (58–83)	<0.001
**Age > 65 year**	53 (42%)	67 (52%)	83 (66%)	<0.001
**BMI (kg/m^2^)**	26 (24–29)	27 (24–30)	26 (24–30)	0.404
**Women**	44 (35%)	39 (31%)	35 (28%)	0.514
**Hypertension**	63 (50%)	74 (58%)	81 (65%)	0.051
**Diabetes**	26 (21%)	29 (23%)	44 (35%)	0.016
**Hypercholesterolemia**	48 (38%)	48 (38%)	67 (53%)	0.013
**Family history of CAD**	31 (24%)	31 (24%)	22 (18%)	0.333
**Current smoking**	55 (43%)	32 (25%)	31 (25%)	0.001
**CV history**				
**Chronic coronary syndrome**	36% (28%)	54 (42%)	43 (34%)	0.067
**Prior MI**	16 (13%)	12 (9%)	20 (16%)	0.284
**CABG**	0 (0%)	0 (0%)	6 (5%)	0.002
**PCI**	18 (14%)	13 (10%)	16 (13%)	0.612
**ASCVD**	32 (25%)	20 (16%)	46 (37%)	0.001
**Chronic renal failure**	7 (6%)	5 (4%)	9 (7%)	0.518
**Congestive heart failure**	2 (2%)	0 (0%)	1 (1%)	0.364
**Admission data**				
**Time to admission (min)**	148 (93–440)	153 (89–383)	170 (80–329)	0.729
**HR (b/min)**	77 (63–88)	78 (65–90)	78 (70–91)	0.024
**SBP (mmHg)**	143 (128–168)	147 (126–170)	134 (114–165)	0.209
**SBP (mmHg)**	89 (77–96)	87 (74–100)	79 (65–100)	0.113
**LVEF (%)**	60 (50–60)	58 (50–60)	50 (45–55)	<0.001
**LVEF < 40%**	5 (4%)	10 (8%)	16 (13%)	0.029
**STEMI**	64 (50%)	73 (57%)	70 (56%)	0.520
**Anterior wall location**	11 (9%)	23 (18%)	40 (32%)	<0.001
**GRACE risk score**	128 (105–155)	140 (112–161)	150 (133–178)	0.028
**Revascularization**				
**PCI**	88 (70%)	123 (96%)	88 (70%)	<0.001
**CABG**	0 (0%)	1 (1%)	29 (23%)	<0.001
**In-hospiral events**				
**CV death**	2 (2%)	1 (1%)	9 (7%)	0.006
**Heart failure**	5 (4%)	7 (6%)	15 (12%)	0.030
**Recurrent MI**	1 (1%)	1 (1%)	3 (2%)	0.430

ASCVD: atherosclerotic cardiovascular disease; PCI: percutaneous coronary intervention; CABG: coronary artery bypass graft; CAD: coronary artery disease; BMI: body mass index; STEMI: ST segment elevation myocardial infarction; LVEF: left ventricular ejection fraction; SBP: systolic blood pressure; DBP: diastolic blood pressure; HR: heart rate.

**Table 2:** Biological data. N(%) or median(IQR).

		SYNTAX Score		
	Tertile 1	Tertile 2	Tertile 3	*p*-Value
	N = 125	N = 128	N = 125	
**OPG, pg/mL**	962 (782–1497)	1240 (870–1707)	1464 (1011–2129)	<0.001
**OPG >1080 pg/mL**	46 (23%)	70 (35%)	82 (41%)	<0.001
**Creatinine (μmol/L)**	75 (61–92)	77 (68–91)	82 (69–97)	0.002
**GFR** **(mL/min/1.73 m^2^)**	93 (77–101)	85 (71–97)	78 (59–95)	<0.001
**Total cholesterol (mmol/L)**	5.11 (4.59–6.08)	5.42 (4.51–6.21)	5.06 (3.89–5.99)	0.094
**HDL cholesterol (mmol/L)**	1.23 (1.04–1.58)	1.32 (1.03–1.55)	1.27 (1.00–1.50)	0.577
**LDL cholesterol (mmol/L)**	3.20 (2.38–3.82)	3.29 (2.50–4.11)	3.05 (2.07–3.88)	0.151
**Triglycerides (mmol/L)**	1.34 (0.89–2.10)	1.53 (1.02–2.16)	1.40 (0.98–2.00)	0.518
**Blood glucose (mmol/L)**	7.1 (5.9–8.8)	6.9 (5.8–8.4)	7.1 (6.2–9.0)	0.016
**CRP (mg/L)**	4.3 (2.9–8.9)	3.0 (2.9–9.9)	4.1 (2.9–10.2)	0.027
**Ntpro-BNP (pg/mL)**	135 (50–1015)	183 (80–1120)	511 (104–1829)	<0.001
**Troponine Ic peak (μg/L)**	30.5 (8.2–78.8)	22.0 (4.5–93.8)	34.5 (4.7–157.5)	0.246

OPG: osteoprotegerin; Nt-proBNP: N-terminal proBrain Natriuretic peptide; CRP: C-reactive protein; LDL: low density lipoprotein; HDL: high density lipoprotein.

**Table 3:** Multivariate logistic regression analysis for the prediction of CAD extent (last SYNTAX Tertile) (OR: odds ratio; CI: confidence interval).

	OR (95% CI)	*p*
**OPG > 1080 pg/mL**	2.10 (1.29–3.49)	0.003
**Hypercholesterolemia**	1.69 (1.08–2.89)	0.019
**Prior CAD**	1.47 (1.11–4.56)	0.018
**GFR < 60 mL/min/1.73 m^2^**	1.99 (1.20–3.05)	0.002

GFR: glomerular filtration rate.

## Data Availability

Supporting data may be found in internal archives.
